# Heart failure in Southern Sweden (HISS): a cross-sectional analysis of primary care patients’ characteristics and physicians’ adherence to guideline-directed medical therapy

**DOI:** 10.1136/bmjopen-2025-101178

**Published:** 2025-11-04

**Authors:** Karin Svensson Söderberg, Lo Kronestedt Lundevall, Thomas Lindow, Oscar Ö Braun, J Gustav Smith, Kristina Sundquist, Veronica Milos Nymberg

**Affiliations:** 1 Center for Primary Health Care Research, Department of Clinical Sciences, Lund University, Malmö, Sweden; 2 Regional Department of Competence in Family Medicine and Primary Health Care, Region Kronoberg, Växjö, Sweden; 3 Pulmonary Medicine, Allergology and Palliative Medicine, Department of Clinical Sciences Lund, Lund University Faculty of Medicine, Lund, Sweden; 4 Research and Development, Region Kronoberg, Växjö, Sweden; 5 Department of Medicine, Växjö Central Hospital, Region Kronoberg, Växjö, Sweden; 6 Cardiology, Department of Clinical Sciences, Lund University and Skåne University Hospital, Lund, Sweden; 7 Department of Molecular and Clinical Medicine, Institute of Medicine, Gothenburg University, Gothenburg, Sweden; 8 Wallenberg Center for Molecular Medicine and Lund University Diabetes Center, Lund University, Lund, Sweden; 9 University Clinic Primary Care, Skåne University Hospital, Region Skåne, Malmö, Sweden

**Keywords:** Heart failure, Cross-Sectional Studies, Primary Care, Guideline Adherence

## Abstract

**Objectives:**

We aimed to describe clinical and diagnostic characteristics of primary care patients with heart failure and physicians’ adherence to guideline-directed medical therapy (GDMT) for treating chronic heart failure.

**Design:**

Cross-sectional study based on baseline data from the prospective primary care-based study Heart failure in Southern Sweden (HISS).

**Setting:**

Patients with heart failure were included from 20 primary healthcare centres in the southernmost region of Sweden (Skåne).

**Participants:**

Between 2020 and 2023, patients were included in HISS, resulting in a total of 587 participants. Of these, 558 patients (95% of the HISS participants) had available data on left ventricular ejection fraction and were included in this study. Adult patients aged 18 years or older diagnosed with heart failure (International Classification of Diseases, 10th Revision codes I50, I11.0, I42, I43) were considered eligible for inclusion in HISS. Community-dwelling patients with assisted care were excluded.

**Primary and secondary outcomes:**

The primary outcome measures were distribution of heart failure subtypes and prescribed medications. The secondary outcomes were temporal trends in GDMT and the association between physicians’ adherence to GDMT and clinical characteristics of patients, using logistic regression models.

**Results:**

Heart failure with preserved ejection fraction (HFpEF) was the most prevalent subtype (42%), followed by mildly reduced (30%) and reduced ejection fraction (HFrEF, 28%). Among patients with HFrEF, 20% were prescribed the recommended GDMT according to the European Society of Cardiology (ESC) 2021 guidelines, which consisted of a renin-angiotensin system inhibitor, a beta-blocker, a mineralocorticoid receptor antagonist and a sodium-glucose 2 inhibitor. We observed no significant change in the prescribing trends for the quadruple therapy in patients with HFrEF when comparing the 2 years before and after the publication of the ESC 2021 guidelines. Similarly, we observed no association between patient characteristics and the prescription of GDMT according to ESC 2021 for patients with HFrEF.

**Conclusion:**

HFpEF was the most prevalent subtype, with conclusive and recent echocardiography data among two-thirds of the cohort. Temporal trends in prescription patterns showed no appreciable increase in the use of GDMT for HFrEF during the two years following guideline publication compared with the two preceding years. These findings indicate a need for inclusion of primary care patients as a basis for intensified medical recommendations and implementation strategies.

**Trial registration number:**

NCT04129658.

**STRENGTHS AND LIMITATIONS OF THIS STUDY**

- This is the first study in a primary care setting to describe diagnostics and treatment characteristics according to the updated European Society of Cardiology 2021 guidelines, in a cohort of patients with heart failure and known ejection fraction.
- The patients were recruited from various primary healthcare centres with high sociodemographic diversity, thus increasing the generalisability of the results.
- All patients in the study had undergone echocardiography, ensuring the accuracy of the heart failure diagnoses.
- There is uncertainty regarding the distribution of different subtypes based on ejection fraction at inclusion, as the echocardiography was conducted at varying times prior to inclusion. Since only the latest echocardiography result was analysed, the proportion of patients with improved ejection fraction remains unknown.
- As the clinicians made decisions based on the available echocardiography results, the findings mirrored primary care practice in general.

## Introduction

 A heart failure diagnosis confers a poor prognosis, with all-cause mortality risk for all heart failure reported to be as high as 35% the first year after diagnosis and more than 60% at 5 years, regardless of whether the ejection fraction is reduced or preserved.^1 2^ Primary healthcare has an important role in Sweden for diagnosing and treating patients with chronic heart failure.^3 4^ However, an earlier Swedish register study has indicated deficient adherence in primary care compared with previous practice guidelines from cardiology societies.^5^ Significant regional differences in the management of heart failure within the whole healthcare system in Sweden have also been reported.^6^

In Sweden, where healthcare is government-funded and available for all residents, there is no national target regarding the percentage of patients with heart failure who should be prescribed the new guideline-directed medical therapy (GDMT) recommended in the European Society of Cardiology (ESC) 2021 guidelines. The new GDMT consists of either an ACE inhibitor (ACE-I) or an angiotensin receptor-neprilysin inhibitor (ARNI) or an angiotensin-receptor blocker (ARB), together with a beta-blocker, a mineralocorticoid receptor antagonist (MRA) and a sodium-glucose cotransporter 2 inhibitor (SGLT2 inhibitor). In addition, GDMT for heart failure is based on the left ventricular ejection fraction derived from echocardiography.^7^ Although some previous reports have suggested low utilisation of echocardiography in primary care, it remains unclear what proportion of patients in primary care undergo echocardiography in the current era, and thus to what extent the primary care heart failure population consists of heart failure with reduced ejection fraction (HFrEF), mildly reduced ejection fraction (HFmrEF) and preserved ejection fraction (HFpEF).^1 8^

There is therefore a need to explore whether information regarding patients with heart failure in primary care can provide important observations for achieving optimal adherence to GDMT. In the current study, we aimed to describe clinical and diagnostic characteristics of primary care patients with heart failure and physicians’ adherence to GDMT for treating chronic heart failure.

## Methods

### Study design

The Heart failure in Southern Sweden (HISS) was a prospective study in primary healthcare that sought to (1) determine adherence to GDMT in primary care and (2) evaluate the effectiveness of a case-based, cardiologist-supervised educational programme to improve GDMT adherence. The current report is a cross-sectional study that describes the findings related to the first general aim of HISS and is based on data collected at baseline.

### Study setting

Between 2020 and 2023, all 166 primary healthcare centres in the southernmost region of Sweden (Skåne) were invited to participate in the HISS study. A pilot study at one primary healthcare centre was conducted in 2020 with the goal of including 50 patients and studying the feasibility of the recruitment method. Due to the onset of the COVID-19 pandemic in early 2020, recruitment was stopped after the inclusion of 24 patients. However, as the feasibility was deemed to be good, the main study was launched in 2021 with no further modifications made to the recruitment method. Information meetings were held with the primary care managers employed by Region Skåne, and those who were interested in participating received a personal visit with additional information from the project leader. Each healthcare centre that agreed to participate and recruit patients for the study designated a research assistant responsible for patient recruitment and data collection. The research assistant was either a registered nurse or a resident physician. Recruiting bias was addressed by encouraging the participating primary care centres to recruit patients with heart failure in need of blood tests and an annual visit to the physician for follow-up. The research group did not provide any additional information to physicians or patients about GDMT at baseline recruitment, although such information may have been available through regional dissemination channels outside the project. The healthcare providers and the enrolled patients were aware that information about prescribed medication would be collected and analysed, and that the focus of the study was adherence to guidelines.

An initial sample size of 850 patients was planned, based on the primary outcome of the intervention study investigating potential medication changes after the treatment conference. As the recruitment in the main study was also delayed by the COVID-19 pandemic, we decided to end the inclusion of patients in December 2023. In total, 587 patients were recruited from 20 participating primary healthcare centres—both public and private, serving both urban and rural communities. The delay due to the global COVID-19 pandemic presented the opportunity to also monitor the adherence in primary care of the new ESC 2021 guidelines,^7^ which were presented and published in Sweden in the programme for cardiovascular diseases within the national system for knowledge-driven management in healthcare in January 2022.^9^

### Study participants

Adult patients aged 18 years or older diagnosed with heart failure (International Classification of Diseases, 10th Revision (ICD-10) codes I50, I11.0, I42, I43) were considered eligible for inclusion in HISS. The research assistant at each centre identified eligible patients and sent letters to assess their interest in participating in the study. Furthermore, patients with hypertrophic cardiomyopathy (ICD-10 codes I42.1 and I42.2) were excluded, as well as community-dwelling patients with assisted care. In the current report, all participants from HISS with available data on left ventricular ejection fraction were included.

### Data collection and assessment

Data regarding pharmacological treatment, echocardiography results, comorbidities, date for heart failure diagnosis and New York Heart Association (NYHA) classification were obtained from the electronic medical records and transferred to case report forms by the responsible nurse and physician at each participating primary healthcare centre. During the baseline visit, blood samples were drawn, ECGs were recorded and blood pressure, weight and height were measured. Scores on the Care Need Index, a measure of socioeconomic factors at the primary healthcare level,^10^ were collected from a regional statistics register in Region Skåne.

We used the ESC 2021 guidelines when determining whether the prescribed medications—renin angiotensin system inhibitor (RASi), beta-blocker and MRA—were at titration or target doses.^7^ The ESC 2021 guidelines are more comprehensive and describe a wider range of medications compared with local recommendations in Region Skåne. The daily target doses for RASi were as follows: enalapril 20 mg, lisinopril 20 mg, ramipril 10 mg, candesartan 32 mg, losartan 150 mg, valsartan 320 mg and sacubitril/valsartan 194/206 mg. Daily target doses for beta-blockers were bisoprolol 10 mg, carvedilol 50 mg and metoprolol 200 mg. The daily target doses for MRA were 50 mg for both eplerenone and spironolactone. Doses below the target levels were classified as titration doses.

### Statistical analysis

We assessed continuous variables for normality using the Kolmogorov-Smirnov test. All variables were non-normally distributed and are presented as median (IQR). Group differences were tested using the Kruskal-Wallis test. Categorical variables are presented as frequencies and percentages. We used the χ² test to compare proportions between groups. All tests were two-sided, and we considered a p value of <0.05 to be statistically significant. Logistic regression analyses were performed to assess the association between patient-related factors (demographics, NYHA class, comorbidities, heart failure duration, systolic blood pressure and estimated glomerular filtration rate mean (eGFR mean)) and physicians’ adherence to GDMT. Analyses were conducted for the subgroup of patients with HFrEF using three different models. Model 1 accounted for sex and age, in model 2, NYHA classification and heart failure duration were also included and model 3 accounted for the previous factors as well as comorbidities, eGFR mean and systolic blood pressure. The results were reported as ORs with 95% CI. Data were analysed using IBM SPSS Statistics V.29, without the use of additional coding or external scripts.

### Patient and public involvement

Neither patients nor the public were involved in the design, conduct, reporting or dissemination of this research.

### Ethical considerations

The investigation conforms with the principles outlined in the *Declaration of Helsinki*^11^. The project was preregistered in ClinicalTrials.gov, registration number NCT04129658.

## Results

A total of 558 patients, corresponding to 95% of the HISS participants, had available data on left ventricular ejection fraction and were included in this study. The recruited patients were spread across 20 different healthcare centres with a median relative Care Need Index of 0.82 (IQR 0.59–1.76).

### Characteristics of the primary care cohort

The baseline characteristics of the HISS cohort with conclusive echocardiograms are presented in table 1. Only a small subset (16%) of the patients were included in the Swedish Heart Failure Registry (SwedeHF) at baseline, thus highlighting the under-representation of primary care patients in heart failure registers. The median age of included patients was 79 years (IQR 72–84 years), and 42% were women. The median duration of heart failure was 5 years (IQR 2–8 years), and almost half of all patients (47%) were in NYHA II. The most common heart failure subtype was HFpEF (42%), followed by HFmrEF (30%) and HFrEF (28%). For almost one-third of the study participants (n=173, 31%), subtype classification was based on echocardiograms conducted more than 3 years prior to inclusion, and this proportion did not differ significantly between the three subtypes. The most common comorbidity in this cohort was hypertension (79%). Patients with HFrEF exhibited the highest prevalence of diabetes and ischaemic heart disease (p<0.005). Atrial fibrillation was most common in patients with HFmrEF and HFpEF (p<0.001).

**Table 1:** Demographic factors, clinical findings, blood tests, comorbidities and heart failure history across heart failure subtypes

	All (N=558)	HFrEF (n=154)	HFmrEF (n=169)	HFpEF (n=235)	P value	Missing data n (%)
Demographics						
Age, years	79 (72–84)	77 (71–84)	79 (72–83)	79 (74–83)	0.219	–
Female sex	234 (42)	59 (38)	66 (39)	109 (46)	0.191	–
Clinical findings						
BMI, kg/m^2^	27.8 (24.9–32.0)	27.2 (24.8–31.2)	27.4 (24.3–32.8)	28.3 (25.5–32.0)	0.191	44 (8)
SBP, mm Hg	130 (120–145)	130 (120–143)	130 (120–145)	135 (121–149)	0.116	4 (1)
DBP, mm Hg	76 (70–85)	75 (70–85)	79 (70–85)	76 (70–83)	0.624	4 (1)
Heart rate, bpm	69 (61–79)	68 (61–78)	70 (63–81)	69 (60–78)	0.061	7 (13)
Blood tests						
HbA1c, mmol/mol	41 (38–47)	41 (38-51)	41 (38–47)	40 (37–46)	0.024*	9 (2)
FPG, mmol/L	6.1 (5.6–7.1)	6.4 (5.7–7.8)	6.0 (5.5–6.9)	6.1 (5.5–6.9)	0.005*	3 (1)
Cholesterol, mmol/L	4.0 (3.4–4.9)	3.8 (3.3–4.8)	3.8 (3.3–4.7)	4.2 (3.5–5.0)	0.003*	3 (1)
LDL, mmol/L	2.4 (1.8–3.2)	2.2 (1.7–3.1)	2.2 (1.8–3.0)	2.6 (2.0–3.4)	0.004*	3 (1)
NT-proBNP, ng/L	1040 (409–2156)	1084 (474–2428)	1269 (414–2472)	916 (360–1892)	0.174	7 (1)
Creatinine, μmol/L	90 (72–113)	99 (76–127)	89 (73–110)	86 (71–108)	0.001*	3 (1)
eGFR mean, mL/min/1.73 m²	51 (38–62)	47 (33–59)	52 (40–62)	52 (40–62)	0.010*	10 (2)
Medical history						
Atrial fibrillation	309 (55)	61 (40)	104 (62)	144 (61)	<0.001*	–
COPD	93 (17)	25 (16)	32 (19)	36 (15)	0.621	–
Diabetes	198 (36)	71 (46)	57 (34)	70 (30)	0.004*	–
Hypertension	441 (79)	124 (81)	127 (75)	190 (81)	0.331	–
Ischaemic heart disease	220 (39)	78 (51)	71 (42)	71 (30)	<0.001*	–
Heart failure history						
Heart failure duration, years	5 (2–8)	5 (2–10)	5 (2–9)	4 (2–7)	0.410	27 (5)
NYHA						
I	139 (30)	30 (23)	45 (31)	64 (33)		
II	217 (47)	65 (51)	66 (46)	86 (45)	0.459	95 (17)
III	100 (22)	30 (23)	30 (21)	40 (21)		
IV	7 (1)	3 (3)	3 (2)	1 (1)		
Registered in SwedeHF	87 (16)	22 (14)	30 (18)	35 (15)	0.619	4 (1)

Values are presented as median (IQR) or n (%). Statistical differences were tested using the Kruskal-Wallis H test or χ² test.

* p<0.05.

BMI, body mass index; bpm, beats per minute; COPD, chronic obstructive pulmonary disease; DBP, diastolic blood pressure; eGFR, estimated glomerular filtration rate; FPG, fasting plasma glucose; HbA1c, haemoglobin A1c; HFmrEF, heart failure with mildly reduced ejection fraction; HFpEF, heart failure with preserved ejection fraction; HFrEF, heart failure with reduced ejection fraction; LDL, low-density lipoprotein; NT-proBNP, N-terminal pro-B-type natriuretic peptide; NYHA, New York Heart Association; SBP, systolic blood pressure; SwedeHF, Swedish Heart Failure Registry.

### Pharmacological treatment

Baseline data on prescribed pharmacological treatment for patients with heart failure are presented in table 2. In summary, a large proportion of the cohort, as well as among the different subtypes, was prescribed the combination of RASi and beta-blockers. In contrast, a considerably lower proportion were prescribed MRA or SGLT2 inhibitor. Titration doses, rather than target doses, were most commonly prescribed for RASi, beta-blockers and MRA. Target dose was most frequently achieved for RASi (25% of the study population), followed by beta-blockers (15%) and MRA (5%). Out of 477 patients that had been prescribed RASi, 55% (n=262) were prescribed an ACE-I, 40% (n=190) an ARB and 5% were prescribed ARNI (n=23), while two patients were prescribed both an ACE-I and ARB. In the group consisting of patients with HFpEF, only 34 patients (15%) were prescribed an SGLT2 inhibitor (7% of the patients included before 2022 and 16% of the patients included during 2022–2023, p=0.15). In total, 91 patients (16%) were included before 2022, when the updated treatment guidelines became available. There were no statistically significant differences observed between those included before and after 2022 when comparing the distribution of different heart failure subtypes.

**Table 2:** Pharmacological treatment across heart failure subtypes

Medical therapy n, (%)	All (N=558)	HFrEF (n=154)	HFmrEF (n=169)	HFpEF (n=235)	P value
RASi	477 (86)	142 (92)	149 (88)	186 (79)	<0.001*
ACEi target dose	99 (18)	25 (16)	34 (20)	40 (17)	<0.001*
ARB target dose	28 (5)	10 (6)	7 (4)	11 (5)	
ARNI target dose	11 (2)	7 (5)	2 (1)	2 (1)	
ACEi titration dose	160 (29)	46 (30)	56 (33)	58 (24)	
ARB titration dose	147 (26)	40 (26)	41 (24)	66 (28)	
ARNI titration dose	12 (2)	8 (5)	2 (1)	2 (1)	
ACEi/ARB unknown dose	9 (2)	3 (2)	4 (2)	2 (1)	
ACEi+ARB	2 (<1)	0 (0)	1 (<1)	1 (<1)	
Other^1^	9 (2)	3 (2)	2 (1)	4 (2)	
Beta-blockers	476 (85)	144 (94)	145 (86)	187 (80)	<0.001*
Target dose	86 (15)	18 (12)	36 (21)	32 (14)	<0.001*
Titration dose	355 (64)	116 (75)	98 (58)	141 (60)	
Unknown dose	17 (3)	7 (5)	6 (4)	4 (2)	
Other^2^	18 (3)	3 (2)	5 (3)	10 (4)	
MRA	191 (34)	70 (46)	51 (30)	70 (30)	0.003*
Target dose	27 (5)	9 (6)	9 (5)	9 (4)	0.024*
Titration dose	163 (29)	60 (39)	42 (25)	61 (26)	
Unknown dose	1 (<1)	1 (1)	0 (0)	0 (0)	
SGLT2i	131 (24)	55 (36)	42 (25)	34 (15)	<0.001*
Loop diuretic	363 (65)	103 (67)	105 (62)	155 (66)	0.623
Treatment combinations					
RASi+beta-blocker	417 (75)	132 (86)	129 (76)	156 (66)	<0.001*
Number of RASi+beta-blocker+MRA+SGLT2i					<0.001*
0	19 (3)	1 (1)	6 (4)	12 (5)	
1	75 (14)	8 (5)	20 (12)	47 (20)	
2	258 (46)	63 (41)	81 (48)	114 (48)	
3	140 (25)	51 (33)	43 (25)	46 (20)	
4	66 (12)	31 (20)	19 (11)	16 (7)	

Values are presented as n (%).

* p<0.05.

ACEi, ACE inhibitor; ARB, angiotensin II receptor blocker; ARNI, angiotensin receptor-neprilysin inhibitor; HFmrEF, heart failure with mildly reduced ejection fraction; HFpEF, heart failure with preserved ejection fraction; HFrEF, heart failure with reduced ejection fraction; MRA, mineralocorticoid receptor antagonist; Other1, ibesartan; Other2, atenolol or propranolol; RASi, renin-angiotensin system inhibitor; SGLT2i, sodium-glucose cotransporter 2 inhibitor.

### Physicians’ adherence to GDMT in accordance with ESC 2021 for patients with HFrEF

HFrEF had the highest proportion of patients that were prescribed the double combination of RASi and beta-blockers (86%, p<0.001) as well as quadruple therapy, which, in addition, included MRA and SGLT2 inhibitor (20%). Table 3 presents data on prescribed GDMT at baseline, which is categorised by time relative to the 2021 ESC guidelines, comparing data collected prior to 2022 with those from 2022 onwards. There were no significant differences in the proportion of patients receiving the full quadruple combination of RASi, beta-blockers, MRA and SGLT2 inhibitor when comparing those included in 2020–2021 with those in 2022–2023 (p=0.806).

**Table 3:** Prescription of GDMT by inclusion period (2020–2021 vs 2022–2023) in patients with HFrEF

	2020–**2021** (n=22)	2022–**2023** (n=132)	P value
RASi	19 (86)	123 (93)	0.269
Beta-blocker	22 (100)	122 (92)	0.182
MRA	9 (41)	61 (46)	0.644
SGLT2 inhibitor	5 (23)	50 (38)	0.170
Quadruple therapy	4 (18)	27 (21)	0.806

Values are presented as n (%). Statistical differences were tested using χ² test.

GDMT, guideline-directed medical therapy; HFrEF, heart failure with reduced ejection fraction; MRA, mineralocorticoid receptor antagonist; RASi, renin-angiotensin system inhibitor; SGLT2, sodium-glucose cotransporter 2.

In addition, no associations were observed between demographic factors, heart failure duration or comorbidities and the prescription of GDMT according to ESC 2021^7^ for patients with HFrEF (table 4).

**Table 4:** Clinical factors associated with the prescription of GDMT according to ESC 2021^7^ for patients with HFrEF

	Model 1	Model 2	Model 3
Female	1.02 (0.45 to 2.29)	1.03 (0.40 to 2.60)	0.93 (0.33 to 2.63)
Age	1.00 (0.96 to 1.04)	0.99 (0.95 to 1.04)	1.00 (0.94 to 1.07)
NYHA			
II	–	1.06 (0.33 to 3.42)	0.96 (0.28 to 3.34)
III–IV	–	2.27 (0.65 to 7.88)	2.01 (0.53 to 7.58)
Heart failure duration	–	1.00 (0.92 to 1.09)	1.01 (0.92 to 1.11)
Atrial fibrillation	–	–	2.05 (0.78 to 5.40)
COPD	–	–	0.94 (0.25 to 3.50)
Diabetes	–	–	1.59 (0.55 to 4.58)
Hypertension	–	–	1.38 (0.36 to 5.26)
Ischaemic heart disease	–	–	1.51 (0.56 to 4.04)
SBP	–	–	0.98 (0.95 to 1.01)
eGFR mean	–	–	1.00 (0.96 to 1.03)

ORs with 95% CIs from logistic regression models for clinical factors associated with GDMT defined as quadruple therapy consisting of RASi, beta-blocker, MRA and SGLT2i.

COPD, chronic obstructive pulmonary disease; eGFR, estimated glomerular filtration rate; ESC 2021, European Society of Cardiology 2021; GDMT, guideline-directed medical therapy; HFrEF, heart failure with reduced ejection fraction; MRA, mineralocorticoid receptor antagonist; NYHA, New York Heart Association; RASi, renin angiotensin system inhibitor; SBP, systolic blood pressure; SGLT2i, sodium-glucose cotransporter 2 inhibitor.

## Discussion

### Principal findings

In this primary care-based cohort study of patients with heart failure from Southern Sweden, we made several observations with potential implications for adherence to GDMT. Approximately one-third of the study cohort at inclusion had an echocardiogram result older than 3 years. Second, HFpEF was the most common heart failure class in this context, followed by HFmrEF and HFrEF. Third, titration doses, rather than target doses, were most common when RASi, beta-blockers and MRA were prescribed. Fourth, adherence to the standard treatment recommended by the Swedish National Board of Health and Welfare in 2018, consisting of RASi and beta-blockers,^3^ was high for patients with HFrEF. In contrast, the proportion of patients with HFrEF who were prescribed the quadruple combination recommended in the ESC 2021 guidelines^7^ was considerably lower. Finally, we observed no association between patient characteristics and the prescription of GDMT according to ESC 2021^7^ for patients with HFrEF.

### Strengths and weaknesses of the study

The variation across types of primary healthcare centres that included patients, in terms of both public and private healthcare centres, as well as rural and urban areas, with a wide distribution in terms of sociodemographic characteristics (Care Need Index) contributes to increased generalisability to other primary care settings.

All patients in our analysis had undergone echocardiography, which ensures the reliability of the heart failure diagnoses and provides conclusive data about ejection fraction. Among the included patients in the main study, 95% had a conclusive echocardiography result. This is in contrast to the findings in the 2022 Swedish register study, where only 57% were reported to have a conclusive echocardiogram.^1^ While the risk of selection bias must be considered, we believe that including patients with a heart failure diagnosis based on echocardiography is a strength. The large variation in the time gap between echocardiography and study inclusion is a limitation. This gap limits accurate classification based on ejection fraction at baseline; the subtype may have changed during this period of time. Since only the latest echocardiography result was analysed, the proportion of patients with improved ejection fraction is unknown. As the clinicians made decisions based on the available echocardiography results, which mirror primary care practice in general, we believe that the findings are applicable to a large primary care setting, which is a strength of this study.

We found no associations between patient characteristics and the prescription of GDMT according to ESC 2021^7^ for patients with HFrEF. Possible explanations could be that factors of importance were not included in this analysis, that the studied cohort was too small or that it depended on the limited time from the presentation of the new guidelines to clinicians and patient recruitment in the study. Nonetheless, future studies are needed to explore the underlying factors of importance for adherence to the new GDMT for heart failure at healthcare provider, healthcare system and societal levels.

HFpEF, being the most common heart failure subtype, stands in contrast to previous studies that primarily have been based on secondary care patients, in which patients with HFrEF constituted the majority.^12 13^ Our findings are instead more similar to an earlier Swedish primary care study where 82% had undergone echocardiography, and of those, 41% had an ejection fraction ≤40%.^5^ In other previous register-based studies that are based on both primary and secondary care, there is a considerable variation in the proportion of patients with known ejection fraction (20–57%), which is of relevance when interpreting the occurrence of HFrEF that varies between 35% and 70%.^1 8 14^ Most patients included in the HISS study (95%) had a conclusive echocardiogram performed before recruitment in the study. However, in the current study, 31% of the patients had an echocardiography result that was 3 years or older at inclusion. The lack of a recent conclusive echocardiogram, although common in clinical practice, results in uncertainty about heart failure subclassification by ejection fraction. Even if changes in ejection fraction in both directions seem to be common,^15^ a lack of accurate information might have implications for treatment. Improvement of reduced ejection fraction has led to the description of a new heart failure subtype—heart failure with improved ejection fraction, a not yet well-established definition. Our study lacks data on changes in echocardiography over time, but a previous review has shown that the prevalence of improved ejection fraction ranges from 10% to 52% among patients with reduced ejection fraction at baseline.^16^

The burden of comorbidities was high but similar to that reported in the Swedish register-based study covering both primary and secondary care during 2013–2019, where hypertension was the most common comorbidity in heart failure. Atrial fibrillation was most common in patients with HFpEF and HFmrEF, while diabetes was most prevalent among patients with HFrEF.^1^ On the other hand, our results differ partly from the findings of a study based on the SwedeHF between 2000 and 2021, where hypertension was also the most common comorbidity, while other comorbidities, except coronary artery disease, were more frequent in patients with HFpEF.^12^

In our study, a limited number (16%) of patients had a known registration in the follow-up and research database SwedeHeartFailure (Rikssvikt), often made by a nurse specialised in heart failure management at a cardiology clinic. Although the present data do not allow for a definitive conclusion, this finding may reflect that a high proportion of patients have not been followed earlier at a cardiology clinic.

We found high adherence to the 2018 treatment guidelines from the Swedish National Board of Health and Welfare when RASi and beta-blockers were recommended for patients with heart failure. In 2018, the Swedish National Board of Health and Welfare recommended that at least 65% of patients with heart failure should receive beta-blockers and RASi.^3 17^ This target was surpassed among patients with HFrEF and HFmrEF, but was only marginally achieved for those with HFpEF. According to the ESC 2021 guidelines,^7^ a quadruple combination consisting of RASi, beta-blocker, MRA and SGLT-2 inhibitor was recommended for patients with HFrEF, to reduce the risk of heart failure hospitalisation and death. For patients with HFmrEF, the recommendation was less strong. In these cases, RASi, beta-blocker and MRA might be considered. There were no recommendations regarding disease-modifying treatments for patients with HFpEF. From a Swedish perspective, there is currently no new specific national target level for either patients with heart failure or the specific group with HFrEF who should receive the quadruple medication combination. In our study, the proportion of patients with HFrEF receiving GDMT in alignment with the ESC 2021 recommendations^7 9^ was low (20%) and remained unchanged, independent of whether inclusion occurred before or after the changes of ESC guidelines. Although the lack of statistical significance could be influenced by the relatively small subgroup size, the observed potential difference was limited and does not clearly suggest a clinically relevant change in prescribing patterns during the recruitment period.

The dosages of RASi, beta-blockers and MRAs were primarily at titration doses, which is consistent with ESC 2021 guidelines suggesting the prescription of all four recommended medications at low doses rather than fewer medications at higher doses.^7^ However, it cannot be determined whether the low doses have facilitated the prescription of multiple medications or whether it reflects that the medications were not titrated to the maximum tolerated dose. Considering the findings from another Swedish study based on patients with heart failure in a primary care setting and the long-term heart failure registry study conducted in the Netherlands (TITRATE-HF)^5 18^ it is likely that the treatment in this cohort was suboptimal regarding the medication dosages. Even though the TITRATE-HF study reported relatively high use of GDMT, both the use and dosing of GDMT were often suboptimal, and the underlying reasons were unclear. This underscores the need for further optimisation and implementation strategies within heart failure management. This need is further reinforced by evidence from the multinational randomised trial *Safety, tolerability and efficacy of rapid optimization, helped by NT-proBNP testing, of heart failure therapies* (STRONG-HF), which showed that high-intensity care with rapid uptitration of all medications included in GDMT led to better outcomes for patients hospitalised due to acute heart failure.^19^ Importantly, for interventions aimed to improve GDMT adherence, the clinical usefulness depends not only on the intervention’s efficacy. Other factors, such as scalability and adaptation to the clinical setting in which it is applied, have been emphasised in a recent review on digital interventions, but are likely relevant beyond the digital context.^20^

In summary, our study suggests that there are both similarities and important differences between patients with heart failure managed in primary care compared with those in secondary care, consistent with observations reported in a Swedish registry study published in 2025.^4^ This is important to recognise when GDMT is established and implemented in primary care settings. Importantly, we observed no significant change in the prescribing trends for quadruple therapy in patients with HFrEF when comparing the 2 years before and after the publication of guidelines. These findings, considered alongside previously published research highlighting the insufficient time primary care physicians often have to implement multiple new guidelines,^21^ emphasise the relevance of not only issuing strong recommendations, but also considering conceptual tools such as *time needed to treat* to aid clinical decision-making. Such conceptual tools could help shape guideline content and help clinicians prioritise among the recommendations, ultimately supporting improved GDMT adherence in everyday practice among those patients with the highest needs.

## Conclusions and implications for the future

Our study found that HFpEF was the most common subtype in this primary care cohort, while a significant proportion of patients lacked a recent conclusive echocardiogram. Additionally, titration doses rather than target doses were prescribed, and a low proportion of patients with HFrEF were prescribed the ESC 2021^7^ recommended quadruple combination. These findings, together with our results showing no appreciable change in temporal trends in the prescription of the quadruple combination therapy, highlight the need for improvement in the care of primary care patients with chronic heart failure. Integrating primary care research into the broader scientific framework that informs medical recommendations and implementation strategies could represent a potential approach to enhance the management of patients with chronic heart failure in primary care.

## Data Availability

Data are available upon reasonable request.

## References

[1] Davidge J, Ashfaq A, Ødegaard KM (2022). Clinical characteristics and mortality of patients with heart failure in Southern Sweden from 2013 to 2019: a population-based cohort study. BMJ Open.

[2] Tsao CW, Lyass A, Enserro D (2018). Temporal Trends in the Incidence of and Mortality Associated With Heart Failure With Preserved and Reduced Ejection Fraction. JACC Heart Fail.

[3] (2018). Nationella riktlinjer for hjärtsjukvård stöd för styrning och ledning. Report no.: 2018-6-28. https://www.socialstyrelsen.se/publikationer/nationella-riktlinjer-for-hjartsjukvard--stod-for-styrning-och-ledning-2018-6-28/.

[4] Chen X, Pivodic A, Schaufelberger M (2025). Incident heart failure: comparing management and outcome in primary and hospital settings in Western Sweden 2008-2017. ESC Heart Fail.

[5] Giezeman M, Arne M, Theander K (2017). Adherence to guidelines in patients with chronic heart failure in primary health care. Scand J Prim Health Care.

[6] (2024). Låt hjärtat orka längre - nationell utvärdering av vården vid hjärtsvikt 2024. Report no.: 2024-11-9307. https://www.socialstyrelsen.se/publikationer/lat-hjartat-orka-langre--nationell-utvardering-av-varden-vid-hjartsvikt--2024-11-9307/.

[7] McDonagh TA, Metra M, Adamo M (2021). 2021 ESC Guidelines for the diagnosis and treatment of acute and chronic heart failure. Eur Heart J.

[8] Bellanca L, Linden S, Farmer R (2023). Incidence and prevalence of heart failure in England: a descriptive analysis of linked primary and secondary care data - the PULSE study. BMC Cardiovasc Disord.

[9] (2024). Läkemedelsordförandekollegiet i sverige (lok) 3B. Läkemedelsbehandling vid kronisk hjärtsvikt. https://folkhalsaochsjukvard.rjl.se/api/Evolution/pdf/3bb5e23c-b1ae-4e55-adf5-b2f53bdb66a8.

[10] Sundquist K, Malmström M, Johansson SE (2003). Care Need Index, a useful tool for the distribution of primary health care resources. J Epidemiol Community Health.

[11] RICKHAM PP (1964). HUMAN EXPERIMENTATION. CODE OF ETHICS OF THE WORLD MEDICAL ASSOCIATION. DECLARATION OF HELSINKI. Br Med J.

[12] Tomasoni D, Vitale C, Guidetti F (2024). The role of multimorbidity in patients with heart failure across the left ventricular ejection fraction spectrum: Data from the Swedish Heart Failure Registry. Eur J Heart Fail.

[13] Chioncel O, Lainscak M, Seferovic PM (2017). Epidemiology and one‐year outcomes in patients with chronic heart failure and preserved, mid‐range and reduced ejection fraction: an analysis of the ESC Heart Failure Long‐Term Registry. European J of Heart Fail.

[14] Norhammar A, Bodegard J, Vanderheyden M (2023). Prevalence, outcomes and costs of a contemporary, multinational population with heart failure. Heart.

[15] Savarese G, Vedin O, D’Amario D (2019). Prevalence and Prognostic Implications of Longitudinal Ejection Fraction Change in Heart Failure. JACC Heart Fail.

[16] He Y, Ling Y, Guo W (2021). Prevalence and Prognosis of HFimpEF Developed From Patients With Heart Failure With Reduced Ejection Fraction: Systematic Review and Meta-Analysis. Front Cardiovasc Med.

[17] Nationella riktlinjer – målnivåer hjärtsjukvård målnivåer för indikatorer. Report no.: 2015-10-3. https://www.socialstyrelsen.se/publikationer/nationella-riktlinjer--malnivaer--hjartsjukvard---malnivaer-for-indikatorer-2015-10-3/.

[18] Malgie J, Wilde MI, Clephas PRD (2024). Contemporary guideline-directed medical therapy in de novo, chronic, and worsening heart failure patients: First data from the TITRATE-HF study. Eur J Heart Fail.

[19] Mebazaa A, Davison B, Chioncel O (2022). Safety, tolerability and efficacy of up-titration of guideline-directed medical therapies for acute heart failure (STRONG-HF): a multinational, open-label, randomised, trial. Lancet.

[20] DeVore AD, Walsh MN, Vardeny O (2025). Digital Solutions for the Optimization of Pharmacologic Therapy for Heart Failure. JACC Heart Fail.

[21] Johansson M, Guyatt G, Montori V (2023). Guidelines should consider clinicians’ time needed to treat. BMJ.

