# Supplementary material for: Heart failure in Southern Sweden (HISS): a cross-sectional analysis of primary care patients’ characteristics and physicians’ adherence to guideline-directed medical therapy
Source: BMJ Open. 2025 Nov 4;15(11):e101178. doi: 10.1136/bmjopen-2025-101178 (PMC12587902; doi:10.1136/bmjopen-2025-101178)
Supplement: Supplementary file 1 [file bmjopen-2025-101178.reviewer_comments.pdf]

PEER REVIEW HISTORY

BMJ Open publishes all reviews undertaken for accepted manuscripts. Reviewers are asked to complete a checklist review form and are provided with free text boxes to elaborate on their assessment. These free text comments are reproduced below.

ARTICLE DETAILS

Title (Provisional)

Heart failure In Southern Sweden (HISS) – a cross-sectional analysis of primary care patients' characteristics and physicians' adherence to Guideline-directed medical therapy

Authors

Svensson Söderberg, Karin; Kronestedt Lundevall, Lo; Lindow, Thomas; Ö. Braun, Oscar; Smith, J. Gustav; Sundquist, Kristina; Milos Nymberg, Veronica

---

VERSION 1 - REVIEW

**Reviewer**	**1**
**Name**	**Dewi, Ayu Putu Madri**
**Affiliation**	**Amsterdam UMC Location AMC, Epidemiology and Data Science**
**Date**	**12-Mar-2025**
**COI**	**None**

I am an Open Science researcher, who has checked your manuscript for its adherence to a number of Open Science practices.

Below you find the checklist that I used to assess your manuscript for adherence to a number of these practices.

Based on this assessment, I would suggest the following refinements/additions:

1. *Availability of a protocol: OK.*
2. *Pre-registration: Please provide information on whether and where the study was pre-registered.*
3. *Open Materials: N/A.*
4. *Open Data: Please provide a statement about whether and where the data used for this study can be accessed.*
5. *Open Code: N/A.*
6. *Citations: Please provide citations for your materials/data/codes.*
7. *Software and tools used are described: OK.*

8. *Open access publication: OK.*
9. *Reporting checklist provided: OK*
10. *Preprint: Please state explicitly whether and where the preprint of this study can be found.*

**Reviewer**	**2**
**Name**	**Wokhlu, Anita**
**Affiliation**	**University of Florida**
**Date**	**28-May-2025**
**COI**	**None**

This is a cross-sectional prospective study of heart failure in Southern Sweden. It involves 20 primary health centers and 587 patients enrolled in the HISS study. The authors' stated aims are to understand the distribution of HF subtypes in the region and to characterize the medications use. The main finding is that HFpEf is the most common subtype. The predominance of HFpEF is line with larger HF trend studies in Europe although interestingly, a 2024 article citing the Swedish HF registry reported ~20% HFpEF. Regarding the secondary outcome, they found that HFrEF patients were undertreated in references to the 2021 ESC guidelines (although some of the cohort predates the guidelines). The authors claim that their study has added value because all subjects have had an echo (in larger cohorts such as Lindberg et al. 2024 EJHF, this value is missing around 10%) & that these primary care pts are underrepresented in other standard HF registries. It is important to note that the guidelines are described but they focus on HFrEF Rx in 2021 with 2023 update providing revised guidance on HFpEF which is not the focus of this article.

Overall, the cohort is an important one, but there are aspects of the manuscript that require clarification. See below.

Comments.

1. Methods. Was this a purely observational study? Did the health centers and patients receive any information regarding the 2021 guidelines or recommended treatments. Were the enrollees and their providers aware that this was a study looking at compliance with HF recommended treatments?

2. Methods. Furthermore, along these lines, on page 29, the study design packet, there is mention of the joint mutual learning conference. I don't think this is mentioned in the manuscript-- was this done? Is this in the methods? What percentage of treating physicians or physician extenders participated in this intervention?

3. Results. Consider including the heart rate in Table 1 (since the authors chose to include BP).

4. Discussion. Since the guidelines changes in 2021 and the cohort enrollment began in 2020, wouldn't that impact your results? It takes time for guidelines to disseminate. Therefore, during a time of guideline transition, what is the clinical takeaway point regarding secondary outcomes on medication use and trends?

5. Results. What are the reasons why patients were on titration rather target doses or missing medications? Renal failure? BP limitations? Intolerances? Allergy? A failure to prescribe? Given the smaller size of the cohort it would be nice to why patients were not on recommended medications.

4. Results. What proportion of patients were also followed by cardiologists versus primary care?

5. The original design of the study at the end of the paper talks about following medication and HF status over 6 months in the entire cohort but the second half of the manuscript focuses on HFrEF, why did the authors choose to omit these details?

5. Regarding Table 3, this data is only there for HFrEF subgroup, and this group is a relatively small subset. Presumably the 2021 guidelines didn't have guidance on HfPEf at the time, and that wasn't till the update. This required me as the reader to lookup the guidelines which indicates that the manuscript needs appropriate clarification. Somewhere, in the discussion or the results, it should be clear that the 2021 guidelines mainly directed HFrEF quadruple therapy. Similarly, table 4 focuses on HFrEF.

6. Results. Wouldn't hte authors want to show the final meds that the patients were during the study period in all groups rather than just HFrEF?

7. Limitation. It is a limitation that there is not recent echo for this study based on HF subtypes. Please expands limitations section based on any queries or concerns that cannot be addressed.

Reviewer 3

Name J. Schuuring, Mark

Affiliation University of Twente

Date 22-Jul-2025

COI I acknowledge to be a member of a Dutch Cardiovascular Alliance consortium (ADMINISTER II), which is supported by public/not-for-profit organizations (i.e. Sichting Hartcentrum Twente) and partners with independent contributions to the research institute (AstraZeneca & Boehringer Ingelheim). M.J.S. also received grant funding from the Pioneers in Healthcare scheme (University of Twente) and TKI-PPP (Health Holland).**

Svensson Söderberg et al. performed a cross-sectional analysis of primary care patients characteristics and physicians’ adherence to guideline-directed medical therapy. Patients

with heart failure were included from 20 primary health care centres in southernmost region of Sweden (Skåne). Between 2020 and 2023, patients were included in HISS, resulting in a total of 587 participants. The authors concluded that heart failure with preserved ejection fraction (HFpEF) was the most prevalent subtype (42%), followed by mildly reduced (HFmrEF, 30%) and reduced ejection fraction (HFrEF, 28%). Among patients with HFrEF, 20% were prescribed the quadruple drug combination recommended in European Society of Cardiology (ESC) 2021 guidelines, with no change over time. No association was found between clinical characteristics and physicians' adherence to guideline-directed medical therapy for patients with HFrEF. The authors conclude that there was an inertia in the implementation of the updated guidelines directed medical therapy for HFrEF, indicating the need for inclusion of primary care patients as basis for intensified medical recommendations and implementation strategies.

The manuscript is interesting, and the data is valuable. For example, the case-based, cardiologist-supervised educational program is innovative. However, I still have a few questions.

1. How many patients with heart failure were regularly referred to a cardiologist? And which patients were referred and which were not? And how was this supervised educational program chosen?

2. Is heart failure care covered by general practitioners in Sweden? This is not the case in many other countries. Are the results generalizable?

3. Guideline-directed medical therapy may be abbreviated to GDMT in the manuscript.

4. This is a cross-sectional analysis, if I understand correctly, over several years. But are these the same patients? In other words, are we comparing the 2021-2021 baseline with follow-up data from the same patients? And how long was the follow-up? Or are we only looking at the 2020-2021 baseline data and the 2022-2023 baseline data? Because at baseline, it's logical that they have GDMT inertia. Describing inertia is only possible with follow-up, right? Otherwise, you'd describe that there's plenty of room for optimization.

5. Essential references on registrations of GDMT use are missing, like PMID: 38734980 DOI: 10.1002/ejhf.3267

6. Section Conclusions and implications for the future: the solutions could be more concrete. Consider, for example, digital solutions to optimize GDMT, such as PubmedID 39797845 and PubmedID 39217271

VERSION 1 - AUTHOR RESPONSE

Reviewer: 1
Ms. Ayu Putu Madri Dewi, Amsterdam UMC Location AMC
Comments to the Author:

I have some feedback related to open science practices. Please find attached the feedback details.

Manuscript Title: Heart failure In Southern Sweden (HISS) – a cross-sectional analysis of primary care patients characteristics and physicians' adherence to guideline-directed medical therapy
I am an Open Science researcher, who has checked your manuscript for its adherence to a number of Open Science practices. Below you find the checklist that I used to assess your manuscript for adherence to a number of these practices. Based on this assessment, I would suggest the following refinements/additions:

Comment 1. Availability of a protocol: OK.

Comment 2. Pre-registration: Please provide information on whether and where the study was pre-registered.
Response: Yes, the study was pre-registered in Clinical Trials, registration number NCT04129658. We have now clarified this point in the Ethical considerations (page 8, lines 19-21) as follows:

"The project was approved by the Swedish Ethical Review Authority (Dnr 2019-03944) and was pre-registered in ClinicalTrials.gov, registration number NCT04129658."

Comment 3. Open Materials: N/A.

Comment 4. Open Data: Please provide a statement about whether and where the data used for this study can be accessed.
Response: Data will be made available by the research team upon reasonable request, provided all ethical and legal concerns are met. We have added a new subheading, Data availability, along with a corresponding statement after Acknowledgements (page 21, lines 15-16) as follows:

"Data will be made available by the research team upon reasonable request, provided all ethical and legal concerns are met."

Comment 5. Open Code: N/A.

Comment 6. Citations: Please provide citations for your materials/data/codes.
Response: The dataset used in this study was collected by our own research group and is original (not previously published). The analyses were performed using IBM SPSS Statistics 29, without the use of additional coding or external scripts. We have clarified this in the Methods section (page 8, lines 12-13) as follows:

"Data were analysed using IBM SPSS Statistics 29, without the use of additional coding or external scripts."

We have also clarified the text in Data availability (page 21, lines 14-15) as follows:

“The dataset used in this study was collected by our own research group and is original (not previously published).”

Comment 7. Software and tools used are described: OK.

Comment 8. Open access publication: OK.

Comment 9. Reporting checklist provided: OK

Comment 10. Preprint: Please state explicitly whether and where the preprint of this study can be found
Response: This manuscript has not been made available as a preprint.

Reviewer: 2
Dr. Anita Wokhlu, University of Florida
Comments to the Author:
This is a cross-sectional prospective study of heart failure in Southern Sweden. It involves 20 primary health centers and 587 patients enrolled in the HISS study. The authors' stated aims are to understand the distribution of HF subtypes in the region and to characterize the medications use. The main finding is that HFpEF is the most common subtype. The predominance of HFpEF is line with larger HF trend studies in Europe although interestingly, a 2024 article citing the Swedish HF registry reported ~20% HFpEF. Regarding the secondary outcome, they found that HFrEF patients were undertreated in references to the 2021 ESC guidelines (although some of the cohort predates the guidelines). The authors claim that their study has added value because all subjects have had an echo (in larger cohorts such as Lindberg et al. 2024 EJHF, this value is missing around 10%) & that these primary care pts are underrepresented in other standard HF registries. It is important to note that the guidelines are described but they focus on HFrEF Rx in 2021 with 2023 update providing revised guidance on HFpEF which is not the focus of this article.

Overall, the cohort is an important one, but there are aspects of the manuscript that require clarification. See below.
Comments.

Comment 1. Methods. Was this a purely observational study? Did the health centers and patients receive any information regarding the 2021 guidelines or recommended treatments. Were the enrollees and their providers aware that this was a study looking at compliance with HF recommended treatments?
Response: Thank you for these important questions. This paper is a cross-sectional study describing the baseline data collected during the recruitment period between 2020-2023. We have now clarified this in the Abstract (page 2, line 5-6), as follows:

“Design: Cross-sectional study based on baseline data from the prospective primary care-based study Heart failure in Southern Sweden (HISS).”

We have also clarified this in the Method section (page 5, lines 11-15), as follows:

"The Heart failure in southern Sweden (HISS) was a prospective study in primary health care that sought to (1) determine the adherence to GDMT in primary care and (2) evaluate the effectiveness of a case-based, cardiologist-supervised educational program to improve GDMT adherence. The current report is a cross-sectional study that describes the findings related to the first general aim of HISS and is based on data collected at the study baseline."

We have also clarified what information enrollees and their providers received in the Method section (page 6, lines 6-11) as follows:

"The research group did not provide any additional information to physicians or patients about GDMT at baseline recruitment, although such information may have been available through regional dissemination channels outside the project. The healthcare providers and the enrolled patients were aware that information about prescribed medication would be collected and analysed, and that the focus of the study was adherence to guidelines."

Comment 2. Methods. Furthermore, along these lines, on page 29, the study design packet, there is mention of the joint mutual learning conference. I don't think this is mentioned in the manuscript-- was this done? Is this in the methods? What percentage of treating physicians or physician extenders participated in this intervention?
Response: This has now been clarified in the manuscript; see the response to comment 1. The focus of this article is not the effect of the intervention; hence, there is no information regarding the percentage of physicians who participated in the intervention that was carried out after the baseline visits.

Comment 3. Results. Consider including the heart rate in Table 1 (since the authors chose to include BP).
Response: This has now been added to Table 1 (page 10, line 13).

Comment 4. Discussion. Since the guidelines changes in 2021 and the cohort enrollment began in 2020, wouldn't that impact your results? It takes time for guidelines to disseminate. Therefore, during a time of guideline transition, what is the clinical takeaway point regarding secondary outcomes on medication use and trends?
Response: In our study, we found that a low percentage of patients with HFrEF received GDMT in alignment with the ESC 2021 recommendations, irrespective of whether inclusion occurred before or after the changes to the ESC guidelines. This has now been clarified in the Discussion section (page 18, lines 13-19) as follows:

"In our study, the proportion of patients with HFrEF receiving GDMT in alignment with the ESC 2021 recommendations [7, 9] was low (20%) and remained unchanged, independent of whether inclusion occurred before or after the changes of ESC guidelines. Although the lack of statistical significance could be influenced by the relatively small subgroup size, the observed potential difference was limited and does not clearly suggest a clinically relevant change in prescribing patterns during the recruitment period."

Our findings, when interpreted in the context of the previously published report about the insufficient time for primary care physicians to implement all applicable guidelines, highlight the relevance not only of a valid recommendation but also of a conceptual tool like time needed to treat in estimating time requirements, which may both influence guideline content and help clinicians prioritise their work and decisions. The clinical takeaway point has now been clarified in the Discussion section (pages 19, lines 15-25 and page 20, lines 1-2) as follows:

"In summary, our study suggests that there are both similarities and important differences between heart failure patients managed in primary care compared to those in secondary care, consistent with observations reported in a Swedish registry study from 2025 [4]. This is important to recognise when GDMT is established and implemented in primary care settings. Importantly, we observed no significant change in the prescribing trends for quadruple therapy in patients with HFrEF when comparing the two years before and after the publication of guidelines. These findings, considered alongside previously published research highlighting the insufficient time primary care physicians often have to implement multiple new guidelines [21], emphasise the relevance of not only issuing strong recommendations, but also considering conceptual tools such as *time needed to treat* to aid clinical decision-making. Such conceptual tools could help shape guideline content and help clinicians prioritise among the recommendations, ultimately supporting improved GDMT adherence in everyday practice among those patients with the highest needs."

Comment 5. Results. What are the reasons why patients were on titration rather target doses or missing medications? Renal failure? BP limitations? Intolerances? Allergy? A failure to prescribe? Given the smaller size of the cohort it would be nice to why patients were not on recommended medications.
Response: We acknowledge the reviewer's point. The current analysis focuses on describing the proportion of patients receiving recommended medications and their dosing levels. The dataset does not include sufficiently detailed information to determine the clinical reasoning behind individual dose decisions. Understanding the factors that influence dose selection remains an important question for future research.

Comment 6. Results. What proportion of patients were also followed by cardiologists versus primary care?
Response: At the time of recruitment, the main treating physician was based in primary care rather than in cardiology. Some patients may have been followed by a cardiologist at an earlier stage, but this could not be reliably determined from the available data. There were a low proportion of patients that had a known registration in the national heart failure registry SwedeHF. This indicates that most of the patients had not previously been followed by a cardiologist since most registrations in this register are often made by a nurse specialised in heart failure management at a cardiology clinic, where the registration coverage in this registry is high. This is in contrast to primary care, which also can use the register, but where the coverage is much lower in general. This has now been clarified in Discussion (p. 17, lines 22-24) as follows:

“Although the present data do not allow for a definitive conclusion, this finding may reflect that a high proportion of patients have not been followed earlier at a cardiology clinic.”

Comment 7. The original design of the study at the end of the paper talks about following medication and HF status over 6 months in the entire cohort but the second half of the manuscript focuses on HFrEF, why did the authors choose to omit these details?
Response: Thank you for this comment. As we explained in our response to Comment 1, this paper is a cross-sectional study describing the baseline data collected during the recruitment period between 2020-2023. We have also clarified this in Results (page 11, lines 15-16) as follows:

“Baseline data on prescribed pharmacological treatment for patients with heart failure are presented in **Table 2**.”

The specific focus on HFrEF is because the ESC 2021 guidelines only had strong recommendations focusing on GDMT for HFrEF, which has now also been clarified in the Discussion, see response to comment 8.

Comment 8. Regarding Table 3, this data is only there for HFrEF subgroup, and this group is a relatively small subset. Presumably the 2021 guidelines didn't have guidance on HfPEf at the time, and that wasn't till the update. This required me as the reader to lookup the guidelines which indicates that the manuscript needs appropriate clarification. Somewhere, in the discussion or the results, it should be clear that the 2021 guidelines mainly directed HFrEF quadruple therapy. Similarly, table 4 focuses on HFrEF.
Response: Thank you for this valuable observation. The specific recommendations from ESC guidelines 2021 for HFrEF, HFmrEF and HFpEF are now stated in Discussion (page 18, lines 3-13) as follows:

“In 2018, the Swedish National Board of Health and Welfare recommended that at least 65% of patients with heart failure should receive beta-blockers and RASi [3, 17]. This target was surpassed among patients with HFrEF and HFmrEF, but was only marginally achieved for those with HFpEF. According to the 2021 ESC guidelines, a quadruple combination consisting of RASi, beta-blocker, MRA, and SGLT-2 inhibitor was recommended for patients with HFrEF, to reduce the risk of heart failure hospitalisation and death. For patients with HFmrEF, the recommendation was less strong. In these cases, RASi, beta-blocker, and MRA might be considered. There were no recommendations regarding disease-modifying treatments for patients with HFpEF. From a Swedish perspective, there is currently no new specific national target level for either patients with heart failure or the specific group with HFrEF who should receive the quadruple medication combination.”

The recommendations for HFrEF have also been clarified in the Abstract (page 2, lines 21-24) as follows:

“Among patients with HFrEF, 20% were prescribed the recommended GDMT according to the European Society of Cardiology (ESC) 2021 guidelines, which consisted of a renin-

angiotensin system inhibitor, a beta-blocker, a mineralcorticoid receptor antagonist, and a sodium-glucose 2 inhibitor."

Comment 9. Results. Wouldn't hte authors want to show the final meds that the patients were during the study period in all groups rather than just HFrEF?
Response: The medications for all patients (both all and on EF subtype basis) are presented in Table 2. We performed a subgroup analysis of HFrEF to examine changes before and after the 2021 ESC guidelines, as the recommendation for the quadruple therapy applied specifically to this group. This has been clarified in the Discussion, please see the response following comment number 8. We also clarified the Result (page 13, lines 10-15) as follows:

"**Table 3** presents data on prescribed GDMT at baseline, which is categorised by time relative to the 2021 ESC guidelines, comparing data collected prior to 2022 with those from 2022 onwards. There were no significant differences in the proportion of patients receiving the full quadruple combination of RASi, beta-blockers, MRA, and SGLT2 inhibitor when comparing those included 2020-2021 with those included in 2022-2023 (p=0.89)."

We have also clarified the title of Table 3 (page 13, line 17) as follows:

"**Table 3. Prescription of GDMT by inclusion period (2020-2021 vs. 2022-2023) in patients with HFrEF**"

Comment 10. Limitation. It is a limitation that there is not recent echo for this study based on HF subtypes. Please expands limitations section based on any queries or concerns that cannot be addressed.
Response 15: This has now been clarified in Discussion (page 16, lines 4-6) as follows:

"The large variation in the time gap between echocardiography and study inclusion is a limitation. This gap limits accurate classification based on ejection fraction at baseline; the subtype may have changed during this period of time."

Reviewer: 3
Dr. Mark J. Schuuring, University of Twente
Comments to the Author:
Svensson Söderberg et al. performed a cross-sectional analysis of primary care patients characteristics and physicians' adherence to guideline-directed medical therapy. Patients with heart failure were included from 20 primary health care centres in southernmost region of Sweden (Skåne). Between 2020 and 2023, patients were included in HISS, resulting in a total of 587 participants. The authors concluded that heart failure with preserved ejection fraction (HFpEF) was the most prevalent subtype (42%), followed by mildly reduced (HFmrEF, 30%) and reduced ejection fraction (HFrEF, 28%). Among patients with HFrEF, 20% were prescribed the quadruple drug combination recommended in European Society of Cardiology (ESC) 2021 guidelines, with no change over time. No association was found between clinical characteristics and physicians' adherence to guideline-directed medical therapy for patients with HFrEF. The authors conclude that there was an inertia in the implementation of the updated guidelines directed medical therapy for HFrEF, indicating the need for inclusion of primary care patients as basis for intensified medical recommendations and implementation

strategies.

The manuscript is interesting, and the data is valuable. For example, the case-based, cardiologist-supervised educational program is innovative. However, I still have a few questions.

Comment 1. How many patients with heart failure were regularly referred to a cardiologist? And which patients were referred and which were not? And how was this supervised educational program chosen?
Response: Thank you for this valuable comment. All patients recruited in this study were managed in primary care. This manuscript focuses on the first aim of HISS to describe the adherence to recommendations; therefore no data are presented on the second aim that focuses on the potential effects of the treatment conference. We have clarified this in the Methods section (page 5, lines 11-15) as follows:

"The Heart failure in southern Sweden (HISS) was a prospective study in primary health care that sought to (1) determine the adherence to GDMT in primary care and (2) evaluate the effectiveness of a case-based, cardiologist-supervised educational program to improve GDMT adherence. The current report is a cross-sectional study that describes the findings related to the first general aim of HISS and is based on data collected at baseline."

Comment 2. Is heart failure care covered by general practitioners in Sweden? This is not the case in many other countries. Are the results generalizable?
Response: It is correct, management of chronic stable heart failure in Sweden is mainly provided by primary care. This has now been clarified in the Introduction (page 4, lines 7-9), as follows:

"Primary health care has an important role in Sweden for diagnosing and treating patients with chronic heart failure [3, 4]."

Given the varying organisation and resources of primary care and cardiology clinics, our findings are primarily generalisable to primary health care systems in which management of chronic heart failure is undertaken in primary care as well as in cardiology outpatient clinics.

Comment 3. Guideline-directed medical therapy may be abbreviated to GDMT in the manuscript.
Response: Guideline-directed medical therapy has now been abbreviated to GDMT in the manuscript.

Comment 4. This is a cross-sectional analysis, if I understand correctly, over several years. But are these the same patients? In other words, are we comparing the 2021-2021 baseline with follow-up data from the same patients? And how long was the follow-up? Or are we only looking at the 2020-2021 baseline data and the 2022-2023 baseline data? Because at baseline, it's logical that they have GDMT inertia. Describing inertia is only possible with follow-up, right? Otherwise, you'd describe that there's plenty of room for optimization.

Response: Thank you for highlighting the inaccurate use of the term “therapeutic inertia” in our manuscript. We have revised the text to more accurately reflect our study design, which examines changes in prescription patterns over time relative to the publication of updated guidelines, rather than follow-up of the same patients. Accordingly, we have removed inertia and instead refer to this outcome as “temporal trends in prescription patterns” throughout the manuscript. Changes have been made in the Abstract (page 2, line 24 and page 3, lines 1-10) as follows:

“We observed no significant change in the prescribing trends for the quadruple therapy in patients with HFrEF when comparing the two years before and after the publication of ESC 2021 guidelines. Similarly, we observed no association between patient characteristics and the prescription of GDMT according to ESC 2021 for patients with HFrEF.

Conclusion: HFpEF was the most prevalent subtype, with conclusive and recent echocardiography data among two-thirds of the cohort. Temporal trends in prescription patterns showed no appreciable increase in the use of GDMT for HFrEF during the two years following guideline publication compared with the two preceding years. These findings indicate a need for inclusion of primary care patients as a basis for intensified medical recommendations and implementation strategies.”

We have also made changes in the Discussion (page 19, lines 15-25 and page 20, lines 1-2) as follows:

“In summary, our study suggests that there are both similarities and important differences between heart failure patients managed in primary care compared to those in secondary care, consistent with observations reported in a Swedish registry study from 2025 [4]. This is important to recognise when GDMT is established and implemented in primary care settings. Importantly, we observed no significant change in the prescribing trends for quadruple therapy in patients with HFrEF when comparing the two years before and after the publication of guidelines. These findings, considered alongside previously published research highlighting the insufficient time primary care physicians often have to implement multiple new guidelines [21], emphasise the relevance of not only issuing strong recommendations, but also considering conceptual tools such as *time needed to treat* to aid clinical decision-making. Such conceptual tools could help shape guideline content and help clinicians prioritise among the recommendations, ultimately supporting improved GDMT adherence in everyday practice among those patients with the highest needs.”

Comment 5. Essential references on registrations of GDMT use are missing, like PMID: 38734980 DOI: 10.1002/ejhf.3267

Response: Thank you for this valuable suggestion. We have added the reference to the following text in the Discussion section (page 18, lines 20-25 and page 19, lines 1-9) as follows:

“The dosages of RASi, beta-blockers, and MRAs were primarily at titration doses, which is consistent with ESC 2021 guidelines suggesting the prescription of all four recommended

medications at low doses rather than fewer medications at higher doses [7]. However, it cannot be determined whether the low doses have facilitated the prescription of multiple medications or whether it reflects that the medications were not titrated to the maximum tolerated dose. Considering the findings from another Swedish study based on patients with heart failure in a primary care setting and TITRATE-HF study [5, 18], it is likely that the treatment in this cohort was suboptimal regarding the medication dosages. Even though the TITRATE-HF study reported relatively high use of GDMT, both the use and dosing of GDMT were often suboptimal, and the underlying reasons for this were unclear. This underscores the need for further optimisation and implementation strategies within heart failure management. This need is further reinforced by evidence from the multinational randomised trial STRONG-HF, which showed that high-intensity care with rapid up-titration of all medications included in GDMT led to better outcomes for patients hospitalised due to acute heart failure [19]. "

6. Section Conclusions and implications for the future: the solutions could be more concrete. Consider, for example, digital solutions to optimize GDMT, such as PubmedID 39797845 and PubmedID 39217271

Response: Thank you for this valuable suggestion. The PubmedID 39797845 has now been added as a reference to make the solutions more concrete. Rather than recommending a single type of intervention, our focus is on highlighting the key dimensions that potential solutions must address in order to be effective in primary care – namely scalability and adaption to the clinical setting. We have added the following text to the Discussion section (page 19, lines 9-13) as follows:

"Importantly, for interventions aimed to improve GDMT adherence the clinical usefulness depends not only on the intervention's efficacy. Other factors such as scalability and adaptation to the clinical setting in which it is applied have been emphasised in a recent review on digital interventions, but are likely relevant beyond the digital context [20]."

VERSION 2 - REVIEW

**Reviewer**	**3**
**Name**	**J. Schuuring, Mark**
**Affiliation**	**University of Twente**
**Date**	**07-Oct-2025**
**COI**	

All my questions have been answered satisfactorily. I have no further questions.
